# Supplementary material for: Predation and fragmentation portrayed in the statistical structure of prey time series
Source: BMC Ecol. 2009 May 6;9:10. doi: 10.1186/1472-6785-9-10 (PMC2689204; doi:10.1186/1472-6785-9-10)
Supplement: Additional file 2 — Voles and related classes ODDox Documentation. ODDox documentation of the agent-based model (ALMaSS) applied by Hendrichsen et al. The documentation is started by activating main.html. [file 1472-6785-9-10-S2.zip › Vole_ODDox/struct_rotation.html]

ALMaSS ODDox: Rotation Struct Reference

- Main Page
- Related Pages
- Classes
- Files

- Alphabetical List
- Class List
- Class Hierarchy
- Class Members

# Rotation Struct Reference

`#include <croprotation.h>`

List of all members.

|  |  |
| --- | --- |
|  |  |
| Public Attributes | |
| TTypesOfVegetation | CropNum [NoFarmTypes] |
| TTypesOfVegetation | NewCrop [NoFarmTypes][4] |
| int | Percent [NoFarmTypes][4] |

---

## Member Data Documentation

TTypesOfVegetation Rotation::CropNum[NoFarmTypes]

TTypesOfVegetation Rotation::NewCrop[NoFarmTypes][4]

int Rotation::Percent[NoFarmTypes][4]

---

The documentation for this struct was generated from the following file:

- croprotation.h

---

Generated on Thu Jan 22 14:13:46 2009 for ALMaSS ODDox by 
 1.5.6
